# Supplementary figures and images for: Selective culture enrichment and sequencing of feces to enhance detection of antimicrobial resistance genes in third-generation cephalosporin resistant Enterobacteriaceae
Source: PLoS One. 2019 Nov 8;14(11):e0222831. doi: 10.1371/journal.pone.0222831 (PMC6839868; doi:10.1371/journal.pone.0222831)

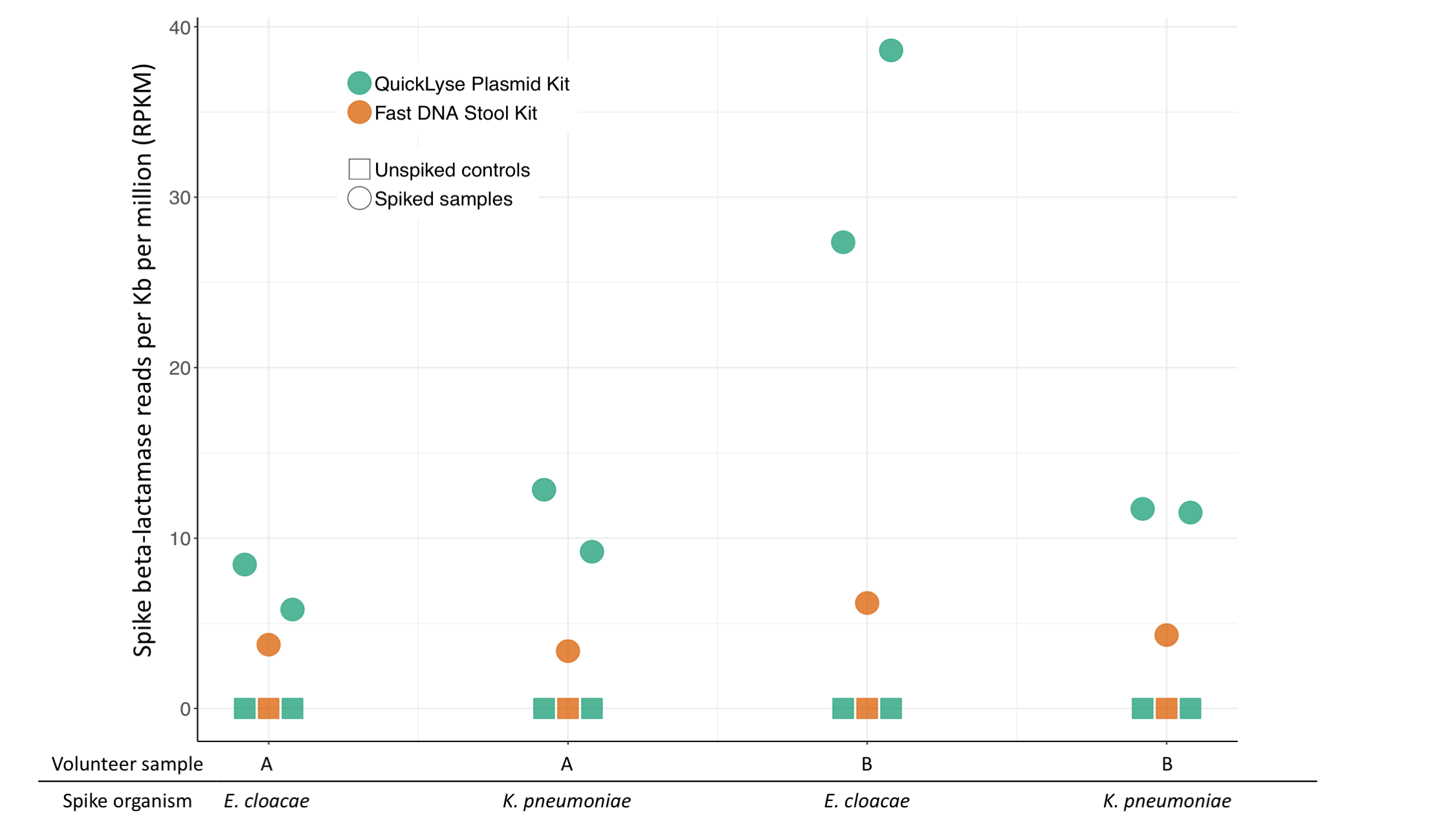

Supplement: S1 Fig — Reads mapping to plasmid-associated beta-lactamases in DNA extracted from fecal samples spiked with 108 CPE/g. Two DNA extraction methods were compared using one of two spike CPE (K. pneumoniae & E. cloacae) and one of two volunteer samples (A & B). QuickLyse extractions were performed in duplicate. (TIF) [file pone.0222831.s001.tif]

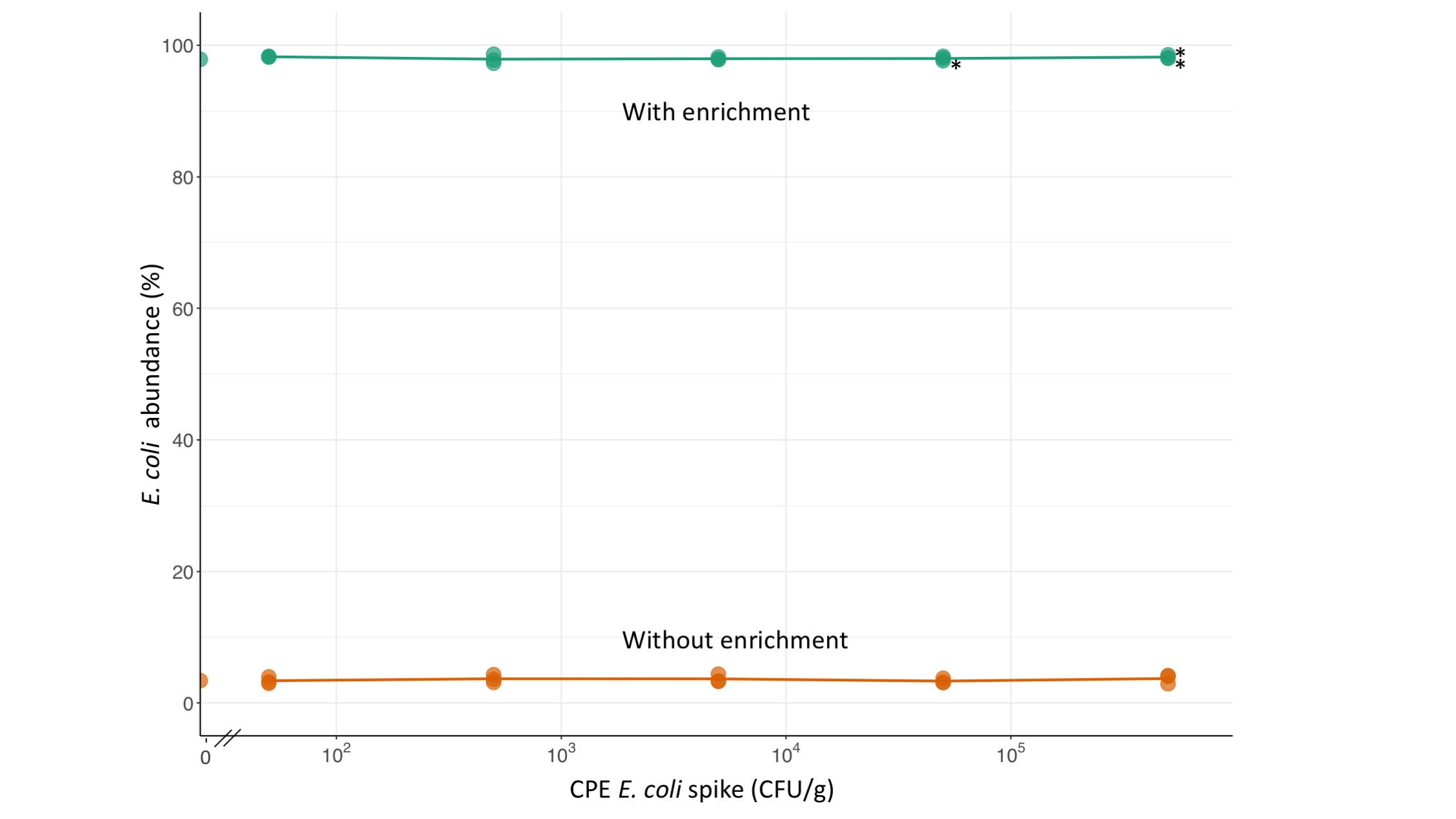

Supplement: S2 Fig — Aliquots of a single fecal sample spiked with NDM-1 E. coli, with or without 6 hours of enrichment in broth containing vancomycin and metronidazole. Note that the sample contained endogenous non-CPE E. coli. Stars indicate the three samples in which a single DNA read mapped to the NDM-1 gene, with no reads mapping to the gene in any other samples. Lines join the arithmetic means of 3 replicates. Unspiked samples are shown on the y-axis. (TIF) [file pone.0222831.s002.tif]

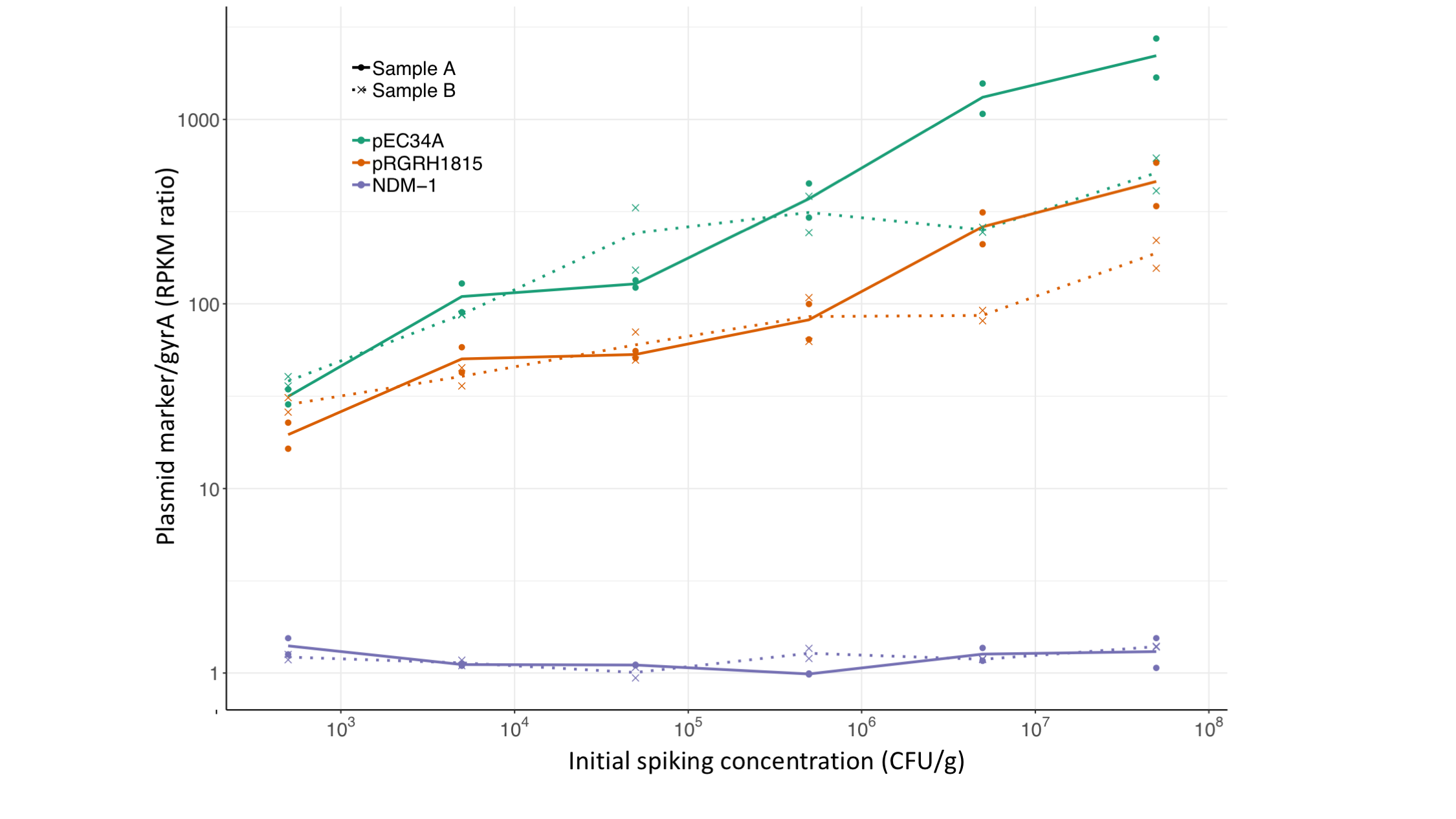

Supplement: S3 Fig — Initial K. pneumoniae spike versus ratio of plasmid-marker to chromosomal gyrA reads. Samples enriched in broth containing cefpodoxime, vancomycin, and metronidazole. Two volunteer samples (A & B) were used. Lines join the geometric means of 2 replicates. (TIF) [file pone.0222831.s003.tif]
